# Mapping the occupational therapy process in response to COVID-19 and long COVID: A scoping review

**DOI:** 10.1177/03080226251384177

**Published:** 2025-11-23

**Authors:** Daniel Cezar da Cruz, Kristine Haertl, George S. Tomlin, Chih-Huang Yu, Oscar Hernández-Lanas, Jess Haigh

**Affiliations:** 1 School of Health, Leeds Beckett University, UK; 2 Department of Occupational Therapy, St. Catherine University, St. Paul, MN, USA; 3 School of Occupational Therapy, University of Puget Sound, Tacoma, WA, USA; 4 Occupational Therapy Program, School of Integrated Health Sciences, University of Nevada, Las Vegas, NV, USA; 5 Department of Occupational Therapy and Occupational Science, Faculty of Medicine, University of Chile, Santiago, Chile; 6 Department of Rehabilitation and Health Professions, Leeds Beckett University, UK

**Keywords:** Occupational therapy, COVID-19, long COVID, evaluation, measurement, outcomes

## Abstract

**Background:**

COVID-19 and long COVID have had an impact worldwide on people’s participation in occupations. Occupational therapists play a role in supporting individuals’ recovery and participation in daily life.

**Objective:**

This present study undertook a scoping review of research on COVID-19 and long COVID to map the occupational therapy process with this population, including evaluation, intervention and outcomes.

**Methodology:**

Three online databases were searched to identify research papers published between 2020 and 2023 from all countries, published in English, Portuguese, or Spanish. From 455 texts, 25 studies were selected for this review.

**Results:**

Studies were conducted across varied healthcare settings, mainly inpatient hospitals. Participants ranged from children to older adults, with adults being the most represented group. Standardised assessments included occupational history, activities, body functions, cognition and emotional regulation. Interventions were educational, compensatory, restorative or acquisitional, with outcomes focused on daily living activities, performance skills and client factors.

**Conclusion:**

Our review underscores the need for more comprehensive documentation of occupational therapy effectiveness, particularly in unpredictable circumstances such as COVID.

## Introduction

COVID-19 (henceforth, COVID) emerged in Wuhan, China, in December 2019 (Zhu et al., 2020) and rapidly evolved into a global pandemic, resulting in substantial mortality, long-term health complications among survivors and significant disruptions to multiple dimensions of daily life. Its ongoing impact is evident in changes to occupational, educational, recreational and social domains, all of which have important implications for population health and well-being. To respond to individual and community needs, occupational therapists provided support services and adopted new ways of delivering occupational therapy (Sinclair, 2021).

In healthcare, the occupational therapy role extended beyond the acute and subacute phases of COVID-19 to include support for individuals with long COVID, a chronic condition following SARS-CoV-2 infection, marked by a continuous, relapsing-remitting or progressive course affecting one or more organ systems for at least 12 weeks (Ely et al., 2024). Distinguishing between ongoing symptomatic COVID (5–12 weeks) and long COVID (12 weeks or more) is crucial, as each stage requires specific clinical and therapeutic approaches (National Health Service – NHS England, 2021).

The COVID pandemic necessitated significant adaptations to the occupational therapy process and modes of service delivery. A survey of 2750 occupational therapists, assistants and students from 100 countries (Hoel et al., 2021), documented the impact of COVID on services, the lack of preparation, new job roles, use of technology, changing work circumstances and/or resource restrictions affecting the occupational therapy process. Within the existing literature, several models of the occupational therapy process have been proposed to guide clinical reasoning and service delivery in diverse contexts. Therefore, for the purposes of this article, there is a need to define the occupational therapy process. In the current scoping review, the occupational therapy process is ‘the client-centred delivery of occupational therapy services. The three-part process includes (1) evaluation and (2) intervention to achieve (3) targeted outcomes and occurs within the purview of the occupational therapy domain’ (American Occupational Therapy Association [AOTA], 2020: 17). It is important to clarify that this framework was chosen as the conceptual model for this scoping review, as its conceptualisation, which includes evaluation, intervention and outcomes, provides a clear and concise framework for categorising the stages of the occupational therapy process represented in the included literature. Alternative models, such as Jennifer Creek’s (2003) process, while comprehensive, adopt a more detailed descriptive approach that encompasses stages such as referral and information gathering, and action plan which were often not reported in sufficient detail across the reviewed articles. Furthermore, given that the majority of the included studies originated from the United States, the AOTA process was considered particularly relevant, as it reflects the predominant framework guiding occupational therapy practice in that context. This alignment facilitated both the classification of findings and the contextual interpretation of the evidence base.

In addition, our Scoping Review considered essential concepts to address the complexity of occupational therapy practice, for example, classifying the approaches into top-down or bottom-up. A top-down or descending approach refers to an Occupation-Centred Practice where assessments and interventions are occupation-based or focused (Fisher and Marterella, 2019), while a bottom-up or ascending approach is focused on performance skills and/or client factors such as emotional aspect, cognitive, physical, sensory, motor, psychological, mental health, amongst other components (Kennedy et al., 2013). Therefore, the objective of our scoping review was to map the occupational therapy process with populations with COVID and long COVID, including evaluation, intervention and outcomes.

## Methodology

This scoping review followed the five stages proposed by Arksey and O’Malley (2005), updated by Peters et al. (2020): (1) Identifying the research question, (2) Identifying relevant studies, (3) Study selection, (4) Charting the data and (5) Collating, summarising and reporting the results using the PRISMA Extension for scoping reviews (PRISMA-ScR; Tricco et al., 2018). A scoping review was a suitable research design to map the occupational therapy practice since this type of study has a range of aims, such as examining the size, variety and sorts of evidence, summarising results of a field of knowledge and identifying gaps to be addressed in future research, amongst other objectives (Tricco et al., 2018). Examining occupational therapy practice during COVID may support future research and workforce practice preparation for global health disasters such as pandemics.

This scoping review was informed by the authors’ prior involvement in an international group of scholars known as the Global Occupational Therapy Think Tank (GOTTT), which was dedicated to critically examining the global occupational therapy response to the COVID pandemic (Almhdawi et al., 2023). The group undertook a review of case studies published by occupational therapists who worked with individuals during the pandemic and subsequently identified the need for a systematic investigation to comprehensively map the occupational therapy process. Moreover, while the evidence from studies related to COVID was widespread, little was investigated about the scope of the occupational therapy process with populations with COVID and long COVID, suggesting the need for the current scoping review. Tricco et al. (2018) recommended stating a clear objective and research question based on acronyms such as Population, Concept and Concept; Setting, Population/Perspective, Intervention, Comparison, Evaluation or Patient, Intervention, Comparison and Outcomes (PICO; Richardson et al., 1995). Therefore, in our research, we considered PICO since we were focused in mapping interventions:

**(a) P-**Population: all populations (children, adolescents, adults and older adults) with COVID or who had COVID and underwent the occupational therapy process as reported in the literature. We included individuals who had COVID and/or long COVID in order to retrieve a range of articles mapping the continuum of occupational therapy services provided.
**(b) I**-Intervention: all occupational therapy interventions with individuals with COVID or long COVID. These interventions can include solely occupational therapy or occur alongside other healthcare, such as physiotherapy.
**(c) C**-Comparison: Although we considered occupational therapy interventions in all healthcare settings (e.g. intensive care units, inpatient hospitals, outpatient clinics, skilled nursing facilities, primary care, home health, amongst others), we did not aim to compare interventions because healthcare is organised in different ways worldwide, affecting occupational therapy service delivery. Instead of comparing, we aimed to describe the diversity of occupational therapy interventions.
**(d) O-**Outcomes: We considered all outcomes of the occupational therapy intervention documented in the articles through standardised measures and non-standardised measures to illustrate the quantitative and qualitative outcomes of the intervention.

Thus, the key descriptions above supported the formulation of the following research question and objective: What is the occupational therapy process with populations with COVID and/or with long COVID? The objective of our scoping review was to map the occupational therapy process with populations with COVID and long COVID, including evaluation, intervention and outcomes.

## Eligibility criteria

Because COVID was a pandemic, our eligibility criteria comprised articles published from 2020 to 2023 from all countries, published in English, Portuguese or Spanish. We included only articles from peer-reviewed journals as we wanted to map reliable sources. To be included in our review, the articles needed to address the occupational therapy process containing evaluation, intervention and outcomes with individuals of any age, country and healthcare setting who received occupational therapy services because of COVID, including long COVID. References from selected articles were included if they matched the inclusion criteria specified above.

We excluded grey literature, editorials, conference proceedings, magazines and letters to the editors for the reason that these texts do not provide new empirical data, or were not subject to rigorous critique before publication. Systematic reviews or scoping reviews were also excluded to avoid duplication of data. Studies with patients during COVID (not with COVID) and research about occupational therapy service changes during COVID were excluded because they were not focused on the occupational therapy process with clients with COVID or long COVID. Observational studies with no description of interventions were also excluded.

## Information sources

Initially, the first author contacted a librarian from Leeds Beckett University to discuss the research question, objectives and keywords of the scoping review. As recommended by the librarian, three databases were chosen: Scopus, CINAHL and Medline, because these databases comprise a range of publications in healthcare, including occupational therapy journals, as identified in previous research (Cruz et al., 2019). After the search, data were exported to a research personal assistant software, Zotero.Org, where duplicates were removed by the first and last authors.

## Search

Tricco et al. (2018) recommended a full electronic search strategy for at least one database. Our study provides the full search strategy developed by the academic librarian for three databases, date range 2020–2023, to allow replication by further researchers as follows:

1. Scopus: Search with Abstract for all: ‘occupational therapy’ OR ‘occupational therapist’
 AND intervention* OR ‘case study’ OR ‘case studies’ OR ‘case report’
 AND sars-cov-2 OR COVID-19 OR coronavirus OR pandemic OR lockdown OR COVID.
2. CINAHL: Search all fields: ‘occupational therapy’ OR ‘occupational therapist’
 AND intervention* OR ‘case study’ OR ‘case studies’ OR ‘case report’ AND
 sars-cov-2 OR COVID-19 OR coronavirus OR pandemic OR lockdown OR COVID.
3. MEDLINE: Search all fields: ‘occupational therapy’ OR ‘occupational therapist’
 AND intervention* OR ‘case study’ OR ‘case studies’ OR ‘case report’ AND
 sars-cov-2 OR COVID-19 OR coronavirus OR pandemic OR lockdown OR COVID. MeSH search of MEDLINE MeSH headings: (MH ‘Occupational Therapy’) AND (MH ‘COVID-19’).

## Selection of sources of evidence

The first and last authors (DC and JH, respectively) screened the 455 texts by title and abstract to assure reliability of selection according to the eligibility criteria. If the title and the abstract were insufficient, the researchers screened the main body of the text to determine whether the study addressed individuals with COVID or long COVID and if the occupational therapy process was described in the method and/or results/findings of the study. At this stage, both screened the studies and added notes to each title at Zotero.Org, with a reason for exclusion as the example: ‘Not related to patients with COVID or “Focused on educational perspective at university”.

After this, the first and last authors met again, and an electronic folder was created with the final studies included in the scoping review. All authors conducted further screening to select potential articles from the reference list. From 422 (100%) texts, 393 (93%) titles were indicated by the last author as potentially excludable according to the criteria. The principal author confirmed the exclusion of these articles for the following reasons: theoretical paper (e.g. framework or review); not related to occupational therapy interventions; focused on therapists’ perspectives (e.g. survey); focused on occupational therapy education (e.g. students and or staff, practice placements, remote learning, impact on mental health); focused on nurses, physiotherapists, psychologists, doctors; focused on telerehabilitation during COVID (telerehabilitation for conditions other than COVID/long COVID); focused on effects of vaccines or respiratory effect; focused on interventions not occupational therapy specific (e.g. physical activity, music therapy); only described assessments, not interventions, focused on populations with specific conditions and the impact of COVID on therapeutic provision, such as individuals with cancer, autism, multiple sclerosis, diabetes, learning disabilities, children with developmental coordination disorder, dementia, stroke, healthcare workforce mental health and individuals with arthritis.

Studies involving telerehabilitation were not excluded from this review. Our inclusion criteria encompassed telerehabilitation interventions, provided that their focus was specifically on COVID – related practice. Given the breadth of our search strategy, we anticipated that all relevant studies would be captured, including, but not limited to, interdisciplinary and multidisciplinary team approaches, telerehabilitation and other forms of intervention. However, studies were included only if they presented the occupational therapy process, namely, assessment, intervention and outcomes, thereby ensuring alignment with the scope and aims of this review.

## Data charting process

Prior to data charting, all authors – except the last one (librarian) – read all the papers and made annotations to be discussed with all group members. After this, the authors found a consensus about essential information to be extracted from the articles. Data extraction from the articles followed a form created by the investigators. This form was calibrated before being used by all authors filling it out, to check if clarification was needed or if more information should be included for extraction. After testing, authors found it important to describe in brackets of each section of the form, terminologies such as ‘bottom-up’, ‘occupation-focused’, ‘occupation-based’ and the approaches: ‘restorative’, ‘educational’, ‘compensatory’ and ‘acquisitional’. Two reviewers independently charted the data from each article, and if any disagreements were identified, researchers met to seek consensus. Data were presented in a Table with a summary of each study, followed by a description of inductive themes that described the occupational therapy process with individuals with COVID and long COVID.

## Data items and synthesis of results

One approach to presenting data items involves including key information such as the author(s), year of publication, study location, type of intervention and any comparator used (if applicable), duration of the intervention, characteristics of the study populations (both caregiver and care recipient groups), study objectives, methodological design, outcome measures and principal findings (Arksey and O’Malley, 2005). Results are presented in a descriptive narrative synthesis by the following items of the occupational therapy intervention according to settings, populations, assessments, goals and results and interventions, including tables and figures with visual representation as recommended by Tricco et al. (2018).

## Bias and rigour

The first four authors are members of an international community of occupational therapy scholars, the Global Occupational Therapy Think Tank, and had been reviewing occupational therapy case studies on the theme of COVID during the pandemic. Therefore, they were familiar with the theme. Tricco et al. (2018) affirmed that critical appraisal of the sources of evidence is not mandatory for scoping reviews. Therefore, we did not critically appraise the studies.

Reflexivity is fundamental in a scoping review (Mak and Thomas, 2022). The research team engaged in regular meetings throughout the study, incorporating reflexivity as an integral component of the process. This involved critical discussions of potential biases and limitations, including concerns about the overrepresentation of studies from English-speaking countries. To maximise inclusivity and reduce language bias, we considered studies published in multiple languages besides English, specifically Portuguese and Spanish, reflecting the linguistic expertise of the authors of this article. Additionally, one author (GT) was fluent in German. However, no eligible studies from Germany were identified, and another author (JY) was fluent in Mandarin and conducted targeted searches for relevant publications in that language without success. The first author (DC) is also fluent in Portuguese. The fifth author (OHL) also contributed to the screening and data extraction process of papers in the Spanish language. The inclusion of multiple languages was supported by our requirement for at least two reviewers proficient in each language to ensure accurate screening and data extraction. This multilingual approach aimed to capture a broader and more representative range of evidence relevant to the scoping review’s objectives.

Rigour was maintained across all stages of the scoping review through several strategies: involvement of an academic librarian, implementation of a reliable screening process, calibration of the data extraction form, preliminary data extraction conducted by researchers and data analysis involving multiple researchers (triangulation). All authors actively contributed to the data analysis phase. All studies were screened and had data extraction by two examiners, increasing reliability. Moreover, the study followed the guidelines for scoping review, including PRISMA-ScR, to report the results (Tricco et al., 2018).

## Results

**Figure 1.**
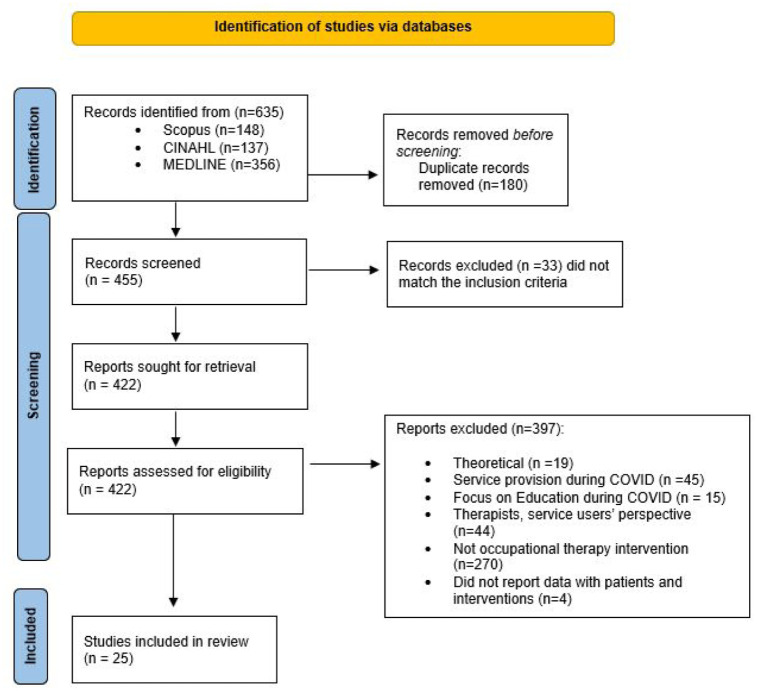
Prisma flow diagram (*n* = 25). Source: Page et al. (2021).

Figure 1 presents the Prisma Flow Diagram.

## Settings

This multi-language scoping review highlighted international settings that shared similar descriptions and titles, yet exhibited some subtle differences, including for instance, the use of ‘Covidarium’ which refers to a special hospital or unit set up to provide care for those with COVID. The articles reviewed spanned the full continuum of care, from intensive care units to outpatient services, with inpatient hospital units emerging as the most frequently identified setting.

## Populations

The articles in this scoping review ranged from single case studies to a large descriptive retrospective cohort study with 432 participants. Males and females were represented across the lifespan. The most frequently represented ages were adults and older adults. All studies included clients in various stages of COVID, many of whom had comorbid conditions. Studies varied in their level of descriptive detail based on the design, number of participants and nature of the study.

## Designs

Of the 25 total included studies, 4 were randomised controlled trials (RCTs; 1 long COVID), 4 were one-group pre–post studies (1 long COVID), 4 were descriptive studies of cohorts receiving OT (2 long COVID), 2 were case series (both COVID) and 11 were individual case studies (2 long COVID).

## Assessments

Table 1 presents the standardised assessments utilised in the studies (*n* = 25).

**Table 1.** Reported standardised assessments.

Categories: ICF/OTPF	Assessment	Frequency	Settings	Citations
Client history	Occupational profile	4	Hospital (acute, inpatient) and SNF	Andrade et al., 2023; Mannion and Sullivan, 2021; Nascimento et al., 2023; Smiley and Reynolds, 2021
	Role checklist	1	Outpatient	Wilcox and Frank, 2021
	Clinical history in medical record	1	Outpatient	Rojas et al., 2022
Activities and participation				
QoL	HR-QoL	1	Telehealth	Calvo-Paniaqua et al., 2022
	WHO QoL BRIEF	1	Community-based hospital	Jung et al., 2023
	Korean Life Balance Inventory	1	Community-based hospital	Jung et al., 2023
Activity limitations				
	AM-PAC	9	Hospital (acute), outpatient	Andrade et al., 2023; Casertano et al., 2021; Coakley et al., 2023; Connors et al., 2022; Mannion and Sullivan, 2021; Meirelles et al., 2021; Walter et al., 2021; Wilcox and Frank, 2021; Wilcox et al., 2021
	Barthel Index	7	Acute-home, inpatient, outpatient, SNF	Colonna et al., 2021; Espino-Tato et al., 2022; Nascimento et al., 2023; Rojas et al., 2022; Smiley and Reynolds, 2021; Trzmiel et al., 2023; Zasadzka et al., 2022
	FIM	4	Hospital (acute, inpatient)	Connors et al., 2022; Nascimento et al., 2023; Trzmiel et al., 2023; Zasadzka et al., 2022
	AMPS	1	Hospital	Christensen et al., 2022
	COPM	1	Community-home	Sclarsky and Kumar, 2021
	CARE tool	2	Hospital (inpatient), SNF	Harrington et al., 2023; Smiley and Reynolds, 2021
	Inpatient rehabilitation facility patient assessment instrument	1	Hospital	Casertano et al., 2021
	Modified Rankin Scale	1	Hospital (acute-inpatient-home)	Colonna et al., 2021
	Lawton IADL scale	1	Outpatient	Wilcox and Frank, 2021
	Functional status score for the intensive care unit	1	Hospital	Coakley et al., 2023
	Fatigue Assessment Scale	1	Hospital (inpatient)	Zasadzka et al., 2022
Body functions/Performance skills				
Balance	6MWT	3	Telehealth, Hospital (acute-inpatient-home)	Calvo-Paniaqua et al., 2022; Colonna et al., 2021; Trzmiel et al., 2023
	Berg Balance Scale	2	Hospital (acute-inpatient-home)	Colonna et al., 2021; Trzmiel et al., 2023
	Tinetti	1	Inpatient	Trzmiel et al., 2023
	Timed up and go	1	Hospital (acute-inpatient-home)	Colonna et al., 2021
	Trunk control test	1	Hospital (acute-inpatient-home)	Colonna et al., 2021
	1-minute sit-to-stand	1	Outpatient	Wilcox and Frank, 2021
Activity tolerance (Dyspnoea)				
	Borg exertion	3	Telehealth, hospital	Calvo-Paniaqua et al., 2022; Luken et al., 2021; Rodrigues et al., 2022
	Dizziness episodes up exertion	1	Outpatient	Wilcox and Frank, 2021
	15-count breathlessness score for activity tolerance	1	telehealth	Luken et al., 2021
	Edmonton symptom assessment	1	Inpatient	Nascimento et al., 2023
Delirium				
	Confusion assessment method	2	Inpatient	Nascimento et al., 2023; Wilcox et al., 2021
	Cornell assessment of paediatric delirium	1	Hospital (acute, inpatient)	Walter et al., 2021
Body structures/Client factors				
Vital signs	BP/HR/RR/SpO_2_	3	Telehealth, hospital (inpatient)	Luken et al., 2021; Rodrigues et al., 2022; Wilcox et al., 2021
Conscious level	Glasgow Coma Scale	1	Outpatient	Rojas et al., 2022
	Richmond Agitation-Sedation Scale	1	Hospital (acute, inpatient)	Walter et al., 2021
Cognition	MoCA	2	Hospital, telehealth	Christensen et al., 2022; Luken et al., 2021
	SLUMS	2	Hospital (acute), telehealth	Connors et al., 2022; Luken et al., 2021
	Lobo mini-mental	1	Elderly Care Home	Espino-Tato et al., 2022
	MMSE	1	Inpatient	Nascimento et al., 2023
	Short blessed test	1	Hospital	Rich et al., 2022
	Frontal assessment battery	1	Hospital (acute-inpatient)	Roldão et al., 2022
Emotional regulation	Beck Depression Inventory II	1	Hospital	Araghi et al. 2022
	Hospital Anxiety and Depression Scale	1	Hospital	Rodrigues et al., 2022
	Patient Health Questionnaire-9	1	Community-based hospital	Jung et al., 2023
	Zung’s Self-Rating Anxiety Scale	1	Community-based hospital	Jung et al., 2023
	Insomnia Severity Index	1	Community-based hospital	Jung et al., 2023
	Multidimensional State Boredom Scale-8	1	Community-based hospital	Jung et al., 2023
	Fear of coronavirus disease	1	Community-based hospital	Jung et al., 2023
Grip and strength	Handgrip strength in manual dynamometer	3	Hospital (acute, inpatient)	Mannion and Sullivan, 2021; Trzmiel et al., 2023; Zasadzka et al., 2022
	medical research council strength score	1	Hospital (acute, inpatient)	Casertano et al., 2021
Others (non-COVID related)	Posture Assessment Scale for Stroke	1	Hospital (acute, inpatient)	Casertano et al., 2021
	Stroke Upper Limb Capacity Scale	1	Hospital (acute, inpatient)	Casertano et al., 2021
	Nine hole peg test	1	Hospital (acute)	Mannion and Sullivan, 2021
	DASH	1	Hospital (acute, inpatient)	Roldão et al., 2022
	Muscle fatigue assessment	1	Inpatient	Zasadzka et al., 2022

AM-PAC: Activity Measure for Post-Acute Care; AMPS: Assessment of Motor and Process Skills; BP: Blood pressure; CARE: Institutional admission care tool; COPM: Canadian Occupational Performance Measure; DASH: Disabilities of the Arm, Shoulder, and Hand questionnaire; FIM: Functional Independence Measure; HR: Heart rate; HR-QoL: Health-related Quality of Life; IADL: Instrumental activities of daily living; ICF: International Classification of Function; MMSE: Mini Mental State Examination; MoCA: Montreal Cognitive Assessment; OTPF: Occupational therapy practice framework; QoL: Quality of life; RR: Respiratory rate; SLUMS: Saint Louis University Mental Status; SNF: Skilled nursing facility; 6MWT: 6-Minute walk test.

Among the 25 studies, 8 were conducted in an acute care setting, 7 in an inpatient setting, 1 in a long-term acute care setting, 1 in a skilled nursing facility (SNF), 1 in an elder care home, 2 in an outpatient setting, 3 in a community-based setting and 2 in a telehealth setting. Standardised assessments employed in these settings focused on different aspects of occupational performance/level of activity and participation, performance skills/body functions and client factors/body structures (see Table 1).

Overall, studies conducted in the acute care setting addressed general activity limitations, vital signs or delirium. Those in long-term care settings (long-term acute, inpatient, SNF, elder care home) primarily addressed activities of daily living (ADL) function and body function/performance skills of balance and activity tolerance. Studies in outpatient and community settings tended to focus more on health-related quality of life (HR-QoL) and emotional regulation.

An analysis of Table 1 reveals a diverse application of standardised assessments across occupational therapy domains, with a notable emphasis on evaluating activity limitations and body functions. The Activity Measure for Post-Acute Care and Barthel Index are the most frequently used tools, appearing in 9 and 7 studies, respectively, predominantly in hospital and outpatient settings. This suggests a strong clinical focus on functional mobility and independence in daily activities. Assessments such as the Functional Independence Measure and CARE tool also feature prominently, reinforcing the prioritisation of functional outcomes in acute and post-acute care. In contrast, tools assessing quality of life (e.g. World Health Organisation Quality of Life-Brief (WHOQoL-BRIEF), HR-QoL) and emotional regulation (e.g. Beck Depression Inventory II, PHQ-9) are less frequently reported, indicating a potential underrepresentation of psychosocial dimensions in standardised assessment practices.

Another trend is the increasing integration of telehealth as a setting for assessment, particularly for tools like the 6-Minute Walk Test, Borg Exertion Scale, and cognitive measures such as Montreal Cognitive Assessment and Saint Louis University Mental Status. This reflects a shift towards remote service delivery, likely accelerated by the COVID pandemic. Additionally, the data show a wide range of assessments used for specific domains such as balance, cognition and emotional regulation, but each with relatively low frequency, suggesting a lack of consensus or standardisation in these areas. The diversity of tools across settings, from acute hospitals to community-based care, not only highlights the adaptability of occupational therapy assessments but also underscores the need for more unified guidelines to ensure consistency and comparability in clinical practice and research.

In one study of telehealth and one study of a community-based setting, quality of life was measured by HR-QoL and the WHOQoL-BREF, respectively. Emotional regulation was mainly addressed in two community-based settings, evaluating depression using Beck Depression Inventory II, anxiety using Zung’s self-rating anxiety scale and insomnia using Insomnia Severity Index.

## Goals and outcomes

Of the 25 studies, 18 (72%) addressed ADL (dressing, bathing, feeding, grooming, toileting and areas of occupation, including sleep, leisure and work), 10 (40%) performance skills (bed mobility, transfers and mobility), 9 (36%) client factors (strength, endurance, range of motion (ROM) and coordination) and 7 (28%) included addressing mental health concerns (anxiety, depression, well-being and quality of life). Three (12%) of the 25 studies did not specify outcomes from the Occupational Therapy Practice Framework (OTPF; AOTA, 2020), but gave general descriptions (e.g. ‘discharged from hospital’).

Of the 25 studies, 6 dealt with people who had long COVID. In these studies, 5 (83%) addressed client factors, 3 (50%) ADL, 2 (33%) performance skills), 2 (33%) mental health and 1 (16%) sleep and work.

Of the eight studies where the application of statistical analysis would have been appropriate (RCTs, one group pre–post studies), all eight did use statistics. In each study, there were at least some positive statistically significant findings. Only one controlled study (Jung et al., 2023), tested the effect of a specific OT approach (‘time use therapy’) and demonstrated a statistically significantly better performance in the experimental group receiving time-use training. The two studies examining the effect of adding robotics to standard rehabilitation protocols (Trzmiel et al., 2023; Zasadzka et al., 2022; on COVID and long COVID, respectively) found no statistically significant difference between the experimental and control groups, although both groups showed improved rehabilitation outcomes to a statistically significant degree. The fourth RCT examined the use of virtual reality for persons with COVID (Rodrigues et al., 2022). Both experimental and control groups showed less fatigue and anxiety. The experimental group also showed decreased shortness of breath and improved well-being.

Since the 25 studies were conducted in 8 different settings (intensive care unit (ICU), acute hospital, rehabilitation hospital, long-term acute hospital, SNF, outpatient clinic, primary care, home/telerehab), there remain many gaps in the literature. As noted above, only one controlled study (Jung et al., 2023) specifically addressed the effectiveness of an occupational therapy approach. The eight studies performed on single groups or cohorts documented meaningful improvement among participants receiving occupational therapy (four of them with statistically significant results; the other four used descriptive quantitative outcomes only, such as percentages). These studies addressed a mix of OTPF sectors (areas of occupation, ADL, performance skills, client factors, including mental health). The lack of controls for subtracting out the effects of self-healing should be noted.

In the 13 case series and individual case studies, covering settings from ICU to primary care, there was generally more comprehensive coverage of relevant OTPF constructs. The 25 individuals studied, however, even if combined into a single group, make up a fairly small sample, especially given the range of settings.

Table 2 summarises the studies’ characteristics.

**Table 2.** Study characteristics (*N* = 25).

Authors	Design	Population	Stages	Setting	Results
Jung et al.	RCT	2-group	COVID	Time-use OT	Ex > Ct in occupational balance, QoL, mental health, *p* < 0.001
Rodrigues et al.	RCT	2-group	COVID	VR education, counselling	Ex and Ct: less fatigue and anxiety; Ex: more well-being, less SOB, ES small to medium
Tzmiel et al.	RCT	2-group	COVID	Rehab vs rehab + robotics	Ex/Ct both stat sig better, no difference between
Zasadzka et al.	RCT	2-group	Long COV	Rehab vs rehab + robotics	Ex/Ct both stat sig better, no difference between
Araghi et al.	Pre–post	1-group	COVID	Online CBT	Less depression stat sig
Calvo-Paniagua et al.	Pre–post	1-group	Long COV	Telerehab exercise programme	Less dyspnoea, more QoL, endurance, stat sig.
Coakley et al.	Pre–post	1-group	COVID	ICU or rehab	ICU-more strength, coordination, balance, activity tolerance, bed mobility, ADL, ambulation, stat sig; Non-ICU-more mobility, stat sig
Harrington et al.	Pre–post	1-group	COVID	Inpatient rehab	Older sub-group better ADL and mobility; younger better mobility; ES 0.7 – 0.9
Espino-Tato et al.	Pre–post	Cohort	Long COV	NR-did include OT	No cognitive impairment from 27 to 67%; functional independence 60 to 67%
Luken et al.	Pre–post	Cohort	COVID	Telerehabilitation	75% had more function; high satisfaction
Nascimento et al.	Pre–post	Cohort	COVID	OT ADL training	86% were discharged from hospital
Rojas-Cardenas et al.	Pre–post	Cohort	Long COV	Multidisciplinary rehab	56 of 122 (46%) fully rehabilitated
Christensen et al.	Case ser	*N* = 11	COVID	NR-did include OT	3/11 more motor and motor process skills; 2/11 independence in ADL
Rich et al.	Case ser	*N* = 3	COVID	NR-used PEOP model	No outcomes reported
Andrade.	Case st	*N* = 1	Long COV	Hospital OT incl mental rehearsal	Better problem-solving and performance awareness
Casertano et al.	Case st	*N* = 1	COVID	Rehab in ICU to Inpatient	Dependence in ADL/mobility to contact guard feeding and oral care
Colonna et al.	Case st	*N* = 1	COV + GBS	OT in ICU to rehab	Independence in ADL except shower, independence in transfers
Connors et al.	Case st	*N* = 1	COV + GBS	OT in long-term acute hospital	LE ROM/coordination, vision WNL; sensation, strength, ADL, transfers better, discharge home
Mannion and Sullivan.	Case st	*N* = 1	COVID	OT in long-term acute hospital	Better seated ADL, transfers, toilet, mobility, self-feeding, strength
Meirelles et al.	Case st	*N* = 1	Lung trans-plant + COVID	OT in ICU to rehab	At discharge: minimum assist transfers, moderate assist ADL, better orientation
Sclarsky and Kumar.	Case st	*N* = 1	COVID	OT consult in primary care	Better meal participation, ADL, cooking; no difference in leisure
Smiley and Reynolds.	Case st	*N* = 1	COVID	OT in SNF	BI 14 to 70 (moderate dependence); MDS CARE and GG 1–2 to 3–5, which is 11 unsafe to 3 unsafe
Walter et al.	Case st	*N* = 1	COVID + MIS-C	OT in PICU to acute hosp	Modified independence in ADL; independence in bed mobility, transfers, short-distance mobility; return to online school
Wilcox and Frank	Case st	*N* = 1	Long COV	OT in outpatient	More activity tolerance, sleep, fatigue management, anxiety/depression coping; return to work as ICU nurse
Wilcox et al.	Case st	*N* = 1	COVID	OT in ICU to rehab	More strength, endurance, mobility, self-care

ADL: Activities of daily living; BI: Barthel Index; Case ser: case series; Case st: case study; CBT: Cognitive behavioural therapy; COV + GBS: COVID and Guillain–Barré syndrome; Ct: Control group; ES: Effect size; Ex: Experimental group; ICU: Intensive care unit; incl: Including; LE ROM: Lower extremity range of motion; Long COV: Long COVID; MDS CARE and GG: Minimum data set care and 3-day admission sections; MIS-C: Multisystem inflammatory syndrome in children; NR: Not reported in article; OT: Occupational therapy; PEOP: Person environment occupation performance; PICU: Paediatric intensive care unit; QoL: Quality of life; RCT: Randomised controlled trial; rehab: Rehabilitation; SNF: Skilled nursing facility; SOB: Shortness of breath; stat sig: Statistical significance; VR: Virtual reality; WNL: Within normal limits.

## Interventions

Interventions included a variety of approaches, grouped into educational, compensatory, restorative and acquisitional. A synthesis of these interventions is presented below:

- **Educational**: actions in this category included teaching self-monitoring and task management strategies (Araghi et al., 2022); using the Dynamic Interactional Cognitive approach to teach strategies (Andrade, 2023); providing health education (Calvo-Paniagua et al., 2022; Christensen et al., 2022; Trzmiel et al., 2023; Zasadzka et al., 2022); providing education on lifestyle modifications and symptom self-management (Wilcox et al., 2021); teaching the time-use intervention approach (Jung et al., 2023); educating on energy conservation and facilitating support groups (Luken et al., 2021; Meirelles et al., 2021; Wilcox et al., 2021); training caregivers on task simplification strategies (Sclarsky and Kumar, 2021); educating nursing staff and family members (Smiley and Reynolds, 2021; Wilcox et al., 2021); educating on sleep/wake cycles and reorientation strategies (Rich et al., 2022; Walter et al., 2021) and instructing on the use of adaptive technology for daily activities (Walter et al., 2021).
- **Compensatory**: actions in this category included the development of structured activity schedules to manage overwhelming tasks (Araghi et al., 2022); utilisation of adaptive equipment (e.g. Ankle–Foot Orthoses (AFOs), adaptive utensils, assistive technology; Casertano et al., 2021; Colonna et al., 2021; Rich et al., 2022); environmental modifications to enhance accessibility, safety and ADL performance (Wilcox et al., 2021); implementation of energy conservation strategies during ADL performance (Connors et al., 2022; Meirelles et al., 2021; Nascimento et al., 2023; Walter et al., 2021; Wilcox et al., 2021); retraining in ADL (Rich et al., 2022) and modifications to mealtime routines and home environments to optimise task execution (Sclarsky and Kumar, 2021).
- **Restorative**: Actions in this category included a programme for strength, endurance, coordination and physical performance (Christensen et al., 2022; Colonna et al., 2021; Connors et al., 2022; Nascimento et al., 2023; Ng et al., 2020; Smiley and Reynolds, 2021; Trzmiel et al., 2023; Wilcox et al., 2021); monitoring mood and cognitive changes during activity (Araghi et al., 2022); interventions in cognitive processing (Connors et al., 2022; Meirelles et al., 2021; Nascimento et al., 2023); prevention of contractures (Walter et al., 2021) and robotic rehabilitation technology for motor function recovery (Trzmiel et al., 2023).
- **Acquisitional**: this category included the training in new cognitive skills for daily activity management (Andrade, 2023; Wilcox et al., 2021); practice with therapists on time-use intervention planning (Jung et al., 2023); retraining in ADL performance, including dressing and mobility tasks (Mannion and Sullivan, 2021; Meirelles et al., 2021); incorporation of technology to enhance IADL (IADL) engagement (e.g. bill payments, household tasks; Rich et al., 2022) and structured training to promote safe transfers and self-care activities (Mannion and Sullivan, 2021; Wilcox et al., 2021).

An analysis of Table 2 reveals distinct patterns in occupational therapy interventions for individuals affected by COVID versus those experiencing long COVID. Interventions for acute COVID cases predominantly focus on short-term functional recovery, often within hospital, ICU, or inpatient rehabilitation settings. These include strength and mobility training, ADL support and psychological interventions such as cognitive behavioural therapy (CBT) and virtual reality education. Studies such as Jung et al. (2023) and Rodrigues et al. (2022) demonstrate statistically significant improvements in occupational balance, mental health and fatigue reduction. The use of telerehabilitation and online platforms also reflects an adaptive response to pandemic constraints, with high satisfaction and functional gains reported (Luken et al., 2021).

In contrast, interventions for long COVID tend to address persistent, multifaceted symptoms such as fatigue, dyspnoea, cognitive impairment and emotional regulation. These are often delivered in community or outpatient settings and involve more holistic, multidisciplinary approaches. For example, Calvo-Paniagua et al. (2022) and Wilcox and Frank (2021) reported improvements in endurance, sleep and return-to-work outcomes. Long COVID studies also show a greater emphasis on rehabilitation continuity and psychosocial support, with outcomes like increased functional independence and coping strategies for anxiety and depression. Notably, while both groups benefited from rehabilitation, long COVID interventions appear more tailored to chronic symptom management and client reintegration into daily life, highlighting the need for sustained and individualised therapeutic models.

## Discussion

Our scoping review aimed to map the occupational therapy process with populations with COVID and long COVID – including evaluation, intervention and outcomes. In our study, we identified a comprehensive overview of the various types of interventions identified across studies, categorised into four approaches: Educational, Compensatory, Restorative and Acquisitional. Educational interventions were widely employed, focusing on teaching strategies for symptom self-management, activity planning and informed decision-making. Compensatory strategies were aimed at adapting the environment, incorporating assistive devices and modifying activities to enhance patient independence. The restorative approach, the most reported, focused on the recovery of physical, cognitive and motor functions through therapeutic exercises and rehabilitation programmes. While less frequent, the acquisitional approach emphasised the use of teaching-learning processes and activity analysis to support individuals in acquiring the specific skills or behaviours required for optimal performance within their environment.

Top-down approaches comprised an occupation-centred practice. To clarify, we tried to group the intervention according to the concepts of occupation-based and focused practice. Occupation-based refers to occupational therapists’ methods of evaluation or intervention, in which the client engages in real, desired occupations for evaluation and/or intervention. The occupation is the base of evaluation or intervention (Fisher and Marterella, 2019) and refers to the ‘immediate’ (versus future) focus of an evaluation or purpose of an intervention on occupation (Fisher and Marterella, 2019: 76), for example focusing on the quality of a person’s occupational performance when dressing, preparing a meal or interacting with co-workers. Occupation-focused describes practice where information about the person, environment and occupation relates closely with occupational performance (Fisher and Marterella, 2019).

We identified a range of interventions mainly focused on basic ADL, but in some studies, the need was clear for a blended approach with bottom-up strategies, including exercises (Connors et al., 2022; Rich et al., 2022). For example, Araghi et al. (2022) utilised CBT to engage patients in performing occupations (occupation-based). They also taught patients how to monitor themselves and evaluate their level of activity. Likewise, Meirelles et al. (2021) focused on ADL training and educational strategies for energy conservation. The case report of Mannio and Sullivan (2021) also combined both occupation-based and focused approaches, with a Spanish 42 year old male engaging in ADL, medication management, simulated violin playing and education. It is important to discuss that while our review draws on studies from diverse countries and in multiple languages, the analysis of how cultural, policy and structural contexts shape occupational therapy practice internationally remains constrained by the lack of sufficient data. Cultural values, healthcare policies and systemic infrastructures can significantly influence the scope, delivery and priorities of occupational therapy, particularly in response to global health challenges such as COVID and long COVID. Differences in healthcare funding models, policy responses to the pandemic and societal perceptions of rehabilitation may lead to distinct approaches in assessment, intervention and service delivery. However, the available literature provided minimal detail on these contextual factors, restricting our ability to explore them in depth. A richer understanding of such influences would not only enhance the comparative international value of this work but also support occupational therapists in adapting their practice across diverse global settings.

Overall, occupation-based interventions involved clients performing the following occupations: walking a dog (Christensen et al., 2022); time use interventions to improve occupational balance (Jung et al., 2023); IADL such as paying phone bills, household tasks, amongst others (Rich et al., 2022). Another particular way to apply occupation-based practice was through assessments of occupational performance, for example, observing a client performing with the aim of assessing occupational difficulties (Andrade, 2023). Nascimento et al. (2023) presented a range of occupation-based interventions such as eating, personal hygiene, functional mobility and dressing/undressing. A case report with an 89-year old female in primary care management focused on the simplification of tasks – meal preparation, changing clothes material/styles for dressing and environmental modification (Sclarsky and Kumar, 2021). In the case study of Wilcox et al. (2021), the occupational therapist utilised a variety of strategies to support the individual’s coping and decrease anxiety such as playing his favourite music, engaging in guided imagery, storytelling from his ICU diary and later guiding self-care occupations.

Occupation-focused approaches included discussion of occupation related to the present and future with a client (Andrade, 2023), guidance for showering (Christensen et al., 2022), activity prescription – task-specific recommendation (Wilcox et al., 2021), energy conservation and peer-group support (Luken et al., 2021). Occupation-focused approaches also appeared in the use of assessments, such as the Canadian Occupational Performance Measure, where clients rate their perceived occupations in terms of performance and satisfaction (Sclarsky and Kumar, 2021). Rich et al. (2022) included at the discharge stage some strategies to encourage participation in daily occupations with in-room activities and ADL to prevent deconditioning. In another study, recalling morning dressing routines and modifying them were strategies utilised to enable dressing and personal hygiene, integrating different energy conservation strategies (Walter et al., 2021).

Studies that include bottom-up approaches focused on a programme divided into physical and cognitive domains (Espino-Tato et al., 2022). Cognitive approaches included, but were not limited to, cognitive processing and delirium management (Meirelles et al., 2021). Studies also reported approaches to improve communication and social skills, increase mood and reframe thoughts (Araghi et al., 2022), and breathing exercises to reduce dyspnoea in individuals with fatigue (Calvo-Paniagua et al., 2022; Colonna et al., 2021). In terms of sensory-motor approaches, studies reported re-education principles including Bobath techniques (Zasadzka et al., 2022), strength, endurance and swallowing (Christensen et al., 2022), balance, sensation and proprioception (Coakley et al., 2023; Colonna et al., 2021), walking tolerance and falls prevention (Sclarsky and Kumar, 2021). Positioning (Ng et al., 2020) and other biomechanical approaches such as using technology also aimed to prevent upper limb complications (Casertano et al., 2021; Connors et al., 2022; Meirelles et al., 2021), hand strength (Roldão et al., 2022), ROM, mobilisation, strengthening (Walter et al., 2021) and weight-bearing exercises (Smiley and Reynolds, 2021), and therapeutic exercises in general (Luken et al., 2021; Mannion and Sullivan, 2021; Rich et al., 2022), including use of robotic-assisted rehabilitation (Trzmiel et al., 2023; Zasadzka et al., 2022) were employed.

## Limitations

Our study has limitations due to the scarcity of documented occupational therapy research in the literature. Despite efforts by the authors to find articles in languages other than English, we recognise that most studies included in this scoping review are in English. Another limitation is that studies were classified according to four approaches that may not capture the full richness of interventions, such as aspects of interpersonal relationships, quality of care, caring, compassion and other qualities of occupational therapy interventions. The lack of standardisation in reporting studies, such as case studies, may also mean that the full scope of what occupational therapists did with their clients is not fully reflected, particularly if they used conceptual models of practice or techniques not reported in the studies.

We acknowledge that our choice of search terms may have limited the comprehensive coverage of the literature, particularly regarding multidisciplinary approaches and the distinction between acute COVID and long COVID. While we did not exclude multidisciplinary interventions, studies without sufficient detail on the occupational therapy process were excluded, as our review’s specific aim was to map the occupational therapy process in populations with COVID and long COVID, including evaluation, intervention and outcomes.

This focus differentiates our work from reviews such as Ng et al. (2024), which examined occupational therapy intervention options for post-COVID symptoms. Although our findings are broadly consistent with Ng et al. (2024), we observed that key areas of occupational therapy practice, such as telerehabilitation, pulmonary rehabilitation and mental health, were underrepresented in the included studies. This thematic gap underscores the need for further research to capture better and expand the scope of occupational therapy interventions for individuals affected by COVID and long COVID.

## Conclusion

Our scoping review mapped the occupational therapy practice for clients with COVID and long COVID, illuminating an array of interventions documented across a range of healthcare settings. The studies focused holistically on ADL, performance skills, client factors, including mental health issues, using both top-down (occupation-based/focused) and bottom-up (biomechanical, sensory-motor and cognitive) approaches. This variety of approaches illustrates the complexity of occupational therapy since interventions combine educational, compensatory, restorative and acquisitional approaches.

We can reflect that occupational therapy practice with this population needs to shift approaches to addressing both physical and psychosocial challenges. Notably, while many studies reported statistically significant improvements in outcomes, the overall evidence base remains limited by small sample sizes, a predominance of case studies and a lack of controlled trials specifically testing these occupational therapy approaches. It is understandable, given the circumstances imposed by the pandemic, particularly the high pressure on healthcare workers and staff shortages, that research activities were likely impacted.

To strengthen the empirical evidence base on the effectiveness of occupational therapy for individuals with COVID or long COVID conditions, a substantial increase in published research is needed, either through more controlled studies across diverse settings, a notable expansion of non-controlled and case study reports, or a combination of both. This scoping review did not identify qualitative studies involving people undergoing OT intervention for either form of COVID. Such studies could also fill an important gap in the evidence network, providing guidance for therapists to work more effectively and strategically in a client-centred manner with their clients affected by COVID.

Finally, to strengthen the evidence base, future research should focus on larger, well-controlled studies across various settings and include qualitative investigations to explore fundamental person-centred experiences, which were missing in the analysed studies. Our review highlights the need for more thorough documentation of occupational therapy effectiveness, especially in unpredictable situations like COVID. This can help develop a strategic plan for occupational therapy practice during natural disasters that is evidence-based on what works well to support individuals’ participation in meaningful occupations in daily life.

**Key findings**

- Occupational therapists utilised a variety of assessments combining bottom-up and top-down approaches to address the needs of individuals with COVID.
- There is a gap in qualitative research capturing individuals’ subjective experiences regarding their treatment during COVID.

**What the study has added**

Occupational therapy interventions, particularly those targeting ADL, cognitive function and mental health, led to meaningful improvements across diverse COVID care settings.
